# Rising to the challenges of research in multiple long-term conditions: health and social care research ecosystem approaches

**DOI:** 10.1136/bmjopen-2024-096206

**Published:** 2025-09-14

**Authors:** Anthony De Soyza, Victoria A Goodwin, Kate Roberts, Jane Masoli, Jacqueline Mathews, John Wilding, Gary Nestor, Peter Bower

**Affiliations:** 1 Newcastle University Population Health Sciences Institute, Newcastle upon Tyne, UK; 2 Ageing and Rehabilitation, University of Exeter, Exeter, UK; 3 NIHR Applied Research Collaboration South West Peninsula, Exeter, UK; 4 MRC Clinical Trials Unit at UCL, Institute of Clinical Trials and Methodology, University College London, London, UK; 5 Ageing and Honorary Consultant Geriatrician, Department of Clinical and Biomedical Science, University of Exeter, Exeter, UK; 6 Health and Care Director and Ageing Lead, NIHR South West Peninsula Research Delivery Network, Exeter, UK; 7 NIHR Research Delivery Network Coordinating Centre, Faculty of Medicine and Health, University of Leeds, Leeds, UK; 8 Department of Cardiovascular and Metabolic Medicine, University of Liverpool Institute of Life Course and Medical Sciences, Liverpool, UK; 9 NIHR Applied Research Collaboration Greater Manchester, University of Manchester, Manchester, UK

**Keywords:** Multimorbidity, Feasibility Studies, Research Design

## Abstract

**Abstract:**

**Objective:**

The purpose of this article is to share insights from the National Institute for Health and Care Research Clinical Research Network (NIHR CRN) in delivering research for Multiple Long Term Conditions (MLTC) and to highlight lessons of wider relevance across the research ecosystem.

**Key reflections:**

Designing health and care systems to be more responsive to the needs of people living with MLTC requires a considerable evidence base. When compared with research focused on a single disease area, research relating to MLTC raises unique considerations at the stages of planning, placing, and delivering research studies. Our article describes challenges associated with MLTC research outcomes and outlines different types of interventions to target MLTC, along with related research delivery considerations. Our article also raises important considerations for placing research in the most appropriate setting and highlights the vital role of robust feasibility studies, informed by the lived experience of patients and carers with MLTC, for ensuring that studies are conducted, recruited to, and completed in a timely and appropriate way.

**Conclusion:**

Increasing the evidence base for the prevention and management of MLTC is a necessity for our health and care system. This presents novel challenges that require collaboration between multiple stakeholders. The UK benefits from a unique research infrastructure, including support for the stages of planning and delivery of health and care research. As the health and care system moves towards bringing care closer to patients and service users, the appropriate selection of the health and care settings and research sites in which to deliver MLTC research, in addition to understanding and removing barriers to recruitment and participation for people with MLTCs, are important considerations to enable us to collectively respond to this challenge.

## Introduction

 The management of patients with multiple long-term conditions (MLTC) is increasingly recognised throughout the world as a major challenge for health and social care systems.^1 2^ The majority of these systems are poorly designed for patients with MLTC, who are often their most frequent users.^3^ This leads to poor user experience, suboptimal outcomes and inefficient use of resources. The prevalence of MLTC varies depending on the specific definition used. In 2004, it was estimated that nearly 31% of a sample of the English primary care population had MLTC and 15% had complex MLTC (C-MLTC: three or more chronic conditions affecting three or more different body systems), increasing to 52.8 % with MLTC and 32.7 % with C-MLTC by 2019.^4^ Comorbidities accumulate as people age, resulting in a high prevalence of MLTC in older people. By 2035, nearly 70% of people aged over 65 years old and over 90% of people aged over 85 years are projected to be living with two or more coexisting diseases.^5^ Globally, MLTC increasingly affects younger people,^6^ with earlier onset associated with deprivation.^4^ The MLTC likely varies across age groups and deprivation levels. The epidemiology of MLTC has been a major initial focus of research, in an attempt to improve the understanding of how different diseases ‘cluster’ within individuals and populations.^7^,^9^ There is also growing evidence that the rate of progression of MLTC is accelerated in areas of greater deprivation as well as women and Black ethnic groups, highlighting the need to include diverse populations^2^ in studies of MLTC.

Designing health and care systems to be more responsive to the needs of people with MLTC will require a considerable evidence base. This necessitates a shift in research approach, from a focus on single diseases, to more inclusive investigation of comorbidity and multimorbidity. At present, the evidence base for MLTC is sparse. For example, the recent Cochrane review on interventions in MLTC included only 17 studies.^10^ Many challenges will have to be overcome to develop the evidence needed to support the health and social care research ecosystem, including upskilling the workforce and ensuring data linkage to capture MLTC. The James Lind Alliance Priority Setting Partnerships^11 12^ have highlighted the importance of making this work patient-focused, with input from multiple stakeholders.

Those attempting to develop the MLTC evidence base recognise the parallel needs to increase the volume of research on MLTC (‘MLTC-focused research’) and also to increase the opportunities for people living with MLTC to participate in research that reflects the real-world population and move away from single disease research with homogenous populations (‘MLTC-inclusive research’).

In England, theNational Institute for Health and Care Research (NIHR) has developed a strategic framework for MLTC.^13^ NIHR both funds research and provides a national research delivery infrastructure through the newly established NIHR Research Delivery Network (NIHR RDN) and its predecessor the NIHR CRN. The NIHR CRN supported national coordination, regional networks and an extensive research delivery workforce. The benefits of the NIHR CRN were emphasised during the COVID-19 pandemic, through success stories, such as the RECOVERY and PANORAMIC trials that delivered rapid, coordinated clinical research at scale in response to urgent needs. Over its tenure, the NIHR CRN supported a range of activities in its response to the challenge of MLTC research, within the context of the NIHR’s Strategic Framework for MLTC. MLTC poses a challenge of a similar scope to COVID-19 and arguably represents another epidemic,^1^ although the nature of the challenges to MLTC research is qualitatively different to those of COVID-19.

In this article, we highlight the experience of the NIHR CRN in reimagining the national research ecosystem to respond better to the challenges of MLTC and to highlight general lessons of relevance. We explore the issues in *planning* MLTC research and *placing* it (in terms of populations, geography and existing health and care systems), in order to promote the *delivery* of high-quality studies to time and target.^11 14 15^

### Planning MLTC research

Feasibility and planning are vital steps in the clinical research process. The inherent complexities of designing and delivering MLTC research bring additional considerations to those of single-condition studies, underlining the importance of timely and robust planning. Within MLTC-focused research, there needs to be recognition of the tension between pragmatic 'real-world' research of complex interventions for complex populations in complex health and care systems compared with research that focuses on highly protocolised interventions in homogenous populations.

MLTC research may seek to detect small effects or may be designed on the ‘non-inferiority’ principle. Such studies are likely to require large sample sizes, necessitating large-scale multisite studies, in which rigour and fidelity to the protocol have to be maintained despite the involvement of many stakeholders. Pragmatic data collection and person-centred approaches are needed to enable the high levels of participation required from service users and care providers.

Studies comparing care pathways, such as the comparison of traditional single specialism care delivery against MLTC polyclinics, require flexibility in routine care systems. Such research topics highlight the need to involve commissioners of health and care in both study design (ie, helping to shape the research question) and research delivery (ie, engaging clinical services to adopt new models of care as a part of research).^15 16^ MLTC care and research will need to be cross disciplinary in nature, with hybrid models of research involving both health and social care teams.^11^ Hybrid research is a broad term, but in this context could particularly apply to a study design where some outcomes are ‘usual health and social care’ endpoints, collected by ‘routine care’ staff with additional study personnel collecting certain data (eg, quality of life measures). Notably, social care research capacity and experience are significantly less than healthcare research and future study designs need to factor this in. Building connections across different disciplines and specialisms will be a key enabler.

Researchers need to consider which outcomes to target in an intervention. This process should give due consideration to what would be meaningful outcomes for patients, health planners and policymakers; these can be challenging to identify across heterogeneous populations with MLTC. Future MLTC research may have to adapt to include composite endpoints in view of this. Calculating the effect sizes of interventions and sample size calculations may be challenging until greater MLTC interventions have been attempted. It is important to consider these outcomes at an early stage in the priority setting processes, as well as during the development of research proposals. The involvement of multiple stakeholders with differing preferences can lead to overcomplicated research protocols, such as large questionnaire burden with repetition between tools or complex protocols that disenfranchise the target populations (eg, regular study visits or complex protocols for measuring activity or diet). A number of potential MLTC research outcome challenges are highlighted in table 1. As MLTC disease burdens are often associated with frailty, deprivation and lower literacy levels, balancing data collection and study complexity needs careful thought and the input of patients and carers in the design process is vital.^4^ A core set of outcomes has been proposed for MLTC and includes both patient and system measures.^17^ The research delivery system will need to both train and coordinate research delivery teams to ensure pragmatic data collection with a minimal burden on study participants, which is deliverable across multiple sites. This needs careful thought to reflect the needs of the patient population, as well as those of health and social care settings. The current pressures on UK primary care and social care services necessitate pragmatic protocol design and meaningful engagement with teams working in these settings. Models of remote consent, data linkage and text-based reporting using mobile phones have all supported recruitment and the collection of outcome measures. Health and social care systems will need to develop methods for overcoming these complexities while maintaining the necessary standards of data protection and information security.

**Table 1:** MLTC research outcome challenges

Type of outcome measure	Considerations for research delivery
Routine health and care data	Mortality, hospital admissions and disease-free survival are simpler datasets to capture versus more ‘routine’ but complex data (eg, COPD exacerbations), which may be variably coded.
Biosamples and biometrics	For biometrics, such as blood pressure, height and weight, or for blood and other biosampling, consideration needs to be given to any difference between local laboratories or other potential sources of measurement variation.
Treatment (or care) burden	Tools currently exist to support these measurements and future development, which allows interactive feedback, may allow multiple time point assessments.^20^
Adverse drug reactions	Particularly important in MLTC as many primary drug licensing studies have few patients with MLTC included; side effects and drug–drug interactions are more common in MLTC.
Dietary intake	Diet-based interventions are complex to deliver at large scale unless person directed and driven.^37^,^39^ There may be challenges in individualised prescriptions, adherence (and its measurement) and sustainability of effort. Measuring change in diet as an outcome may be hampered to a degree by self-reporting bias.
Physical activity	Exercise and rehabilitation-based interventions are complex to deliver at large scale unless person directed and driven. Wearable technologies may assist.^40 41^ There may be challenges in individualised prescriptions, adherence (and its measurement) and sustainability of effort.
Quality of life assessments	Many tools exist. The best tool and the minimally clinical important difference are not clear for each tool. Research suggests that ongoing tool refinement may help.^42^
Health and social care delivery satisfaction; person centredness; care fragmentation	Care fragmentation can be a major challenge for people with MLTC engaging in both care and research.^16 43^ Satisfaction may be widely different when polyclinics or MDT clinics are compared with standard models of care. Person centredness is increasingly recognised as important and measurable.^44^
Mental health, physical health and social interactions	The interaction between mental and physical health makes each comorbidity harder to treat. People with major mental health issues are more likely to have several physical health problems and challenges with engaging in social activities and care.^2 7 45^ If an MLTC intervention involves social interaction, study designs need to consider PPI input to alleviate these challenges
Healthcare and social care utilisation and engagement	In addition, interventions may highlight both health and social care services unrelated to the primary research area but which promote health and/or social care engagement,^46^ for example, home-based nurse-led anxiety counselling leading to signposting to social care support for falls prevention. Measuring this systematically can be challenging.

COPD, chronic obstructive pulmonary disease; MDT, multidisciplinary team; MLTC, multiple long-term conditions; PPI, patient and public involvement.

MLTC research presents numerous methodological and statistical challenges. Designs may need to apply clustering methodology. Studies may need to capture key clinical, social and psychological data to ensure representation of the range of MLTC types (eg, C-MLTC or MLTC that include physical and major mental health comorbidities vs physical health MLTCs alone). Capturing and managing multiple outcomes may be complex and has the potential to give rise to inadvertent recruitment bias requiring sophisticated statistical analysis plans. MLTC research is, therefore, likely to require specific complex trials methodology and statistical input during the design phase, with ongoing monitoring during recruitment. As some interventions may have long-term outcomes, or see a return to baseline behaviours, follow-up may require a systems-wide approach using linked data and remote monitoring or simple follow-up protocols. This may be particularly relevant where study populations live in deprived settings or developing economies where housing and employment stability may be more complex.^18^

Consideration must be given to selection of the best tools to capture global health-related quality of life, which are acceptable to all stakeholders, including people with MLTC and commissioners. Outcomes less traditionally accepted in single-condition studies become more important in MLTC-focused research (eg, social function, mental health, adherence, satisfaction with health and care delivery, adverse outcomes and ‘treatment burden’). Trial funders will need to accept the importance of these outcomes beyond a focus on a single disease-specific outcome (eg, blood pressure control). For example, a study that assesses the role of a ‘care bundle’ (including education, physical activity and diet and medicine optimisation) targeting MLTC in a high-risk ethnic minority group may need biosamples or biometrics (eg, HbA1C, blood pressure, Estimated Glomerular Filtration Rate (eGFR) and lipids), multiple questionnaires (single organ focused plus global health-related quality of life measures), assessments of self-empowerment or health literacy, ‘fidelity to intervention’ data collection, as well as cost effectiveness. Additionally, side effects and treatment burden data need to be collected in a meaningful way.

### Interventions to target MLTC

Some major clusters of MLTC are already recognised^8 19^ and range from population health-level challenges (eg, cardiovascular/mental health/metabolic syndrome/diabetes/obesity/cancer clusters) to numerous other clusters found at lower prevalence. Interventions to target MLTC may simply be the addition of interventions already known to successfully treat single conditions or a second condition, arising in the setting of a first (eg, statin and antihypertensive therapy in the setting of known type II diabetes). A major assumption of modern care is that the best standard of care as recommended in guidelines is deliverable, acceptable and adhered to by health and social care staff and those in their care. In MLTC, this is less likely to be achievable for multiple reasons.^20 21^ Therefore, it is important to acknowledge that some MLTC-focused research will have a strong implementation/health services delivery research component, for example, comparing current (disjointed) care against multidisciplinary clinics, where multiple conditions are optimised according to the best practice in one setting.

Furthermore, it is important to acknowledge that approaches that focus on the optimisation of medicine prescribing (ie, reducing drug treatments/deprescribing)^22^ may be as important as additive approaches in MLTC because of the issue of treatment burden. These studies may, therefore, need non-inferiority designs, as well as effective measurement of treatment burden and person satisfaction.^21 23^

Complex healthcare interventions are widely recognised as more representative of what is needed to better manage MLTC. The updated Medical Research Council Framework for developing and evaluating complex interventions,^24^ among other models, sets clear guidance on how to undertake such research, but may need adaptation to reflect the complexities of MLTC research.^25 26^ Some of the challenges of delivering MLTC interventions are noted in table 2.

### Placing and delivering MLTC-inclusive research—what can a health and care research ecosystem do to improve MLTC research?

There are two major themes that increase our knowledge of interventions relevant to those living with MLTC. First, there is cross-sectoral support and pressure for people with MLTC to be included in more ‘single-disease/single social care need’ research.

Second, there is a need to develop a portfolio of ‘MLTC-focused research’ to answer the questions most relevant to MLTC populations.^11^ In addition, those involved in designing, funding and delivering research must ensure that all research, where appropriate, is ‘MLTC-inclusive’. This is vital to ensure the widest possible benefit to those participating in research and that the results of research are applicable to the range of people typically encountered in routine care.

While the clustering of conditions within individuals has been an early focus of MLTC research, geographic and socioeconomic impacts are also relevant. People from deprived areas are more likely to develop MLTC and to do so at an earlier age than the general population.^7^ These people may also face significant barriers in accessing care and participating in research. There is evidence that the distribution of research is often poorly aligned with areas of greatest need.^27^ Research in deprived areas may raise challenges because of a lack of local infrastructure, distance from centres of research excellence and unsuitability of research protocols and measures for diverse populations. Additional challenges relate to language and cultural barriers and possible prior negative experiences with health and social care. Initially, it may even be more costly and slower to deliver MLTC research, complicating planning for funders. Nevertheless, access to national data and associated digital infrastructure supports the placement of studies in the areas of greatest burden^27^ and to ensure that participation and representation are maximised. Such targeting will need to be complemented by innovations in research delivery, such as data collection, to ensure that ‘research burden’ (such as complex measurement) does not add to the ‘treatment burden’ that participants already face. Innovations to develop and test new methods of recruitment and measurement, including community engagement and ‘research on research’, will be important to ensure that placing research in the areas of high burden is matched by measures that facilitate participation for people with MLTC and the communities in which they live. The resources provided within the NIHR-INCLUDE Frameworks are helpful in this regard.^28 29^

Multiple approaches are needed to help improve access to, participation in and development of MLTC research. The first step is recognising the rising epidemic of MLTC and linking with research funders. MLTC is recognised as a global priority.^30^ Within the UK, there has been significant senior-level ‘buy in’ to these needs.^1 13 31^ This should translate into governmental and charity funding streams supporting MLTC-inclusive and MLTC-focused research. The UK Research and Innovation Strategic Priority Fund outlined MLTC as a key priority.^32^ This has resulted in the commitment to fund MLTC research by the NIHR and Medical Research Council, with cross-cutting research support teams and the generation of communities of practice to support collaborative working to advance MLTC research. The NIHR encourages applications for MLTC research across all of its funding programmes, including Research for Patient Benefit (often pilot studies), Health Technology Assessment (HTA) (evidence synthesis and pragmatic intervention studies) and Efficacy Mechanism Evaluation (early small-scale trials with bench science approaches or ‘piggybacked’ onto an HTA intervention study), as well as through its Global Health Programme. There have also been a number of MLTC-specific research funding opportunities, as well as priority setting exercises through the James Lind Alliance involving public coproduction, which has included a set of priorities for MLTC in older populations.^12 33^ In the USA, the National Institutes of Health have similarly recognised the importance of MLTC research and allocated specific funding.^34^ Similar initiatives have been seen across European funding schemes.

Within the UK, the NIHR CRN supported research delivery across England, with a broad range of National Specialty Groups often with single organ/system interests (eg, respiratory disorders and cardiovascular disease) but also cross-cutting themes (eg, ageing, mental health and infection). The COVID-19 pandemic led to greater cross-disciplinary working, and the NIHR CRN community taking on more strategic roles in research development, leveraging years of experience in delivering studies, to develop pragmatic intervention studies, including MLTC. In addition to cross-disciplinary work by clinicians to prioritise research, there has been a focus on engaging charities (often with a more single organ/system focus) to work together and support public panels to help inform and deliver MLTC research. Key lessons from the success of the RECOVERY trial during the COVID-19 pandemic^35^ included large scale, preferably remote protocol training and embedding interventions into routine care. Recognising this, the NIHR CRN established an MLTC working group, which considered general training needs across the research delivery workforce, as well as opportunities to address such gaps through online learning. This will help to address the gaps in research readiness across various social and healthcare settings. The MLTC working group also interacted with funders to disseminate the needs of MLTC-inclusive and MLTC-focused research. Few people will think of themselves as having MLTC and so the NIHR is exploring a public partner community to help with coproduction and the provision of advice on study design and burden.

A recent scoping review of MLTC interventions in primary care recognised that the key barriers to the delivery of MLTC studies include ‘implementation of the complex interventions’ within a clinical environment and the time required for such changes.^15^ Additionally, there are cross-cutting considerations, such as enabling research staff to access data and engagement of potential participants across a number of sites and settings (eg, primary care, therapy facilities and community settings; as well as hospitals, social care settings and nursing homes). The inclusion of ‘research settings’ as well as ‘specialties’ within the new RDN's health and care leadership structures acknowledges the importance of recognising the differences for research delivery environments along the care pathway (eg, primary care, hospitals, community settings and residential).

Data linkage may be challenging across multiple providers and there are significant complexities to linking and porting person-level data across these systems (eg, tracking the effect of a diabetes treatment regimen, including a physical health intervention, across a nursing home, community rehabilitation programme and measuring hospital admissions, and glycaemic control and treatment adherence). Careful thought and planning will be required to determine the level of training of staff available to support MLTC research and the boundaries between delivery of ‘usual care’ and the conduct of research by members of the care and research teams will need careful thought. Close working with research regulators and ethics committees will be important for minimising barriers to research, and learning from successes and failures is of paramount importance. Such activity should support the upskilling of the research community in grant development and enable the research infrastructure to refine its skills in feasibility assessment for MLTC research.^26^ The establishment of the NIHR RDN sees us enter a new phase in the development of the national research delivery infrastructure. In addition to a continued focus on supporting the successful delivery of high-quality research, the network’s role in increasing capacity and capability to deliver research for the future creates an important opportunity to harness the expertise of its National Specialty and Settings Leadership. These groups will serve as communities of practice, sharing research delivery knowledge for shaping better funding bids and protocols and learning from trials delivery after funding to improve the research delivery environment.

The UK’s unique national infrastructure, with its links between care provision (eg, NHS) and research delivery (eg, NIHR CRN/RDN), provides a unique opportunity to intervene at a number of levels of MLTC research development and delivery. Measuring how research systems improve MLTC-focused research will require monitoring and high-quality data collection (including the accurate identification of studies as being ‘MLTC-focused’ and ‘MLTC-inclusive’). Data can be used to ensure that improvement measures designed to enable MLTC research are having the desired effect on the scope, quality and delivery of that research over time.

Once MLTC research has been developed and delivered, it is important to learn from participants’ experience to inform and improve future studies. The UK-based Patient Research Experience Survey is a nationally standardised annual survey used to collect adults’ and children’s views and experiences of participating in NIHR-supported research and is the largest of its kind in England.^36^ Developed by NIHR CRN, the survey invites dynamic feedback on research and should support improvements in protocols. This will be important in the quest for inclusion of more diverse populations and the capture of multiple outcomes.

**Table 2:** Challenges for research delivery in MLTC

Types of interventions to target people with MLTC	Example	Research ecosystem considerations
Single interventions that improve multiple aspects of an MLTC cluster	Improved blood pressure control may lead to better cardiovascular risk control and minimise falls risk through iatrogenic hypotension.	Arguably already well provided for in current research settings, however, certain readouts will need face-to-face visits and may dramatically increase the cost of the research or exclude the most vulnerable or least mobile groups.
Combined single interventions	Two or more interventions applied aiming to improve comorbid conditions (eg, dual pharmacotherapy to improve cardiovascular and respiratory health (nicotine replacement therapy)).	Polypharmacy, adherence and fidelity to intervention can be challenging, particularly over large-scale studies. Effectiveness may be more readily captured than harms or safety elements.
Multimodal (complex) interventions	Two or more interventions (eg, from differing types of healthcare systems or professionals) applied aiming to tackle conditions simultaneously (eg, postmyocardial infarction care bundle with rehabilitation (exercise, group therapy and education) coupled with a drug therapy (eg, heart failure optimisation or similar) or psychological management of coexistent anxiety and nicotine replacement therapy in COPD.	In addition to the above challenges, longer term follow-up and safety outcomes may need remote data collection or innovative solutions to data linkage.
Health service delivery research approaches	Combining the management of MLTC within a wider system and comparing two care pathways (eg, standard care (multiple single disease-focused clinics) vs a polyclinic).	Researchers may need to work with research delivery staff, commissioners and clinical staff to ensure service redesign (eg, to deliver a polyclinic as a trial intervention and ensure that outcome measures can be started in a timely fashion (and be multisite)).
Health education intervention or self-management programmes	Health education helps people to make informed choices but significant gaps in major areas are acknowledged, for example, Tailored versus generic (eg, smoking advice for pregnant mothers vs generic stop smoking advice). MLTC education may be challenged by various comorbidity patterns and the challenge between comprehensive versus overwhelming information packages. Digital health solutions in MLTC research may become more prevalent.^47^	Fidelity to intervention, access for underserved populations and selection bias may all be additional challenges in a healthcare research ecosystem. Adherence may also be problematic.^48^
Social care interventions	Interventions to facilitate social interaction, attendance at healthcare, group exercise activities or mental health well-being may all be a part of MLTC interventions. Importantly, uptake of such support may be an outcome measure of other interventions and such data are often not captured in current studies.^49^	The boundaries between health and social care are becoming blurred. However, the UK social care research environment is less developed, has multiple providers and a lower level of current research expertise. Online training in research leadership and delivery will allow wider groups to contribute to research.
Health promotion activities	Motivational interviewing, life coaching and patient activation.^50^	Fidelity to intervention, access for underserved populations and selection bias may all be additional challenges in a healthcare research ecosystem.

COPD, chronic obstructive pulmonary disease; MLTC, multiple long-term conditions.

## Conclusions

The COVID-19 pandemic demanded greater flexibility and innovative approaches to research delivery. A whole systems approach led to major breakthroughs. Large-scale simple studies led to innovative changes in practice when this single disease process was targeted in a highly challenging environment. Tackling MLTC research is a necessity for our health and care systems and presents novel challenges that require collaboration between multiple stakeholders to shape, develop and deliver novel studies. Learning from successes and failures will address the current lack of evidence and generate research that will facilitate progress towards more person-centred evidence-based care to transform health and care outcomes. In addition to a focus on promoting and enabling MLTC research, the NIHR Strategic Framework for MLTC^13^ highlights the importance of working with the research community to understand barriers to recruitment and making it easier for people with MLTCs to participate by ensuring that studies are designed inclusively. This is vital for ensuring that research does not unjustifiably exclude people with MLTCs. The establishment of the NIHR RDN, with its core purposes of supporting the delivery of high-quality research that enables the best care for our population and increasing capacity and capability to deliver research for the future, will ensure that the new network continues to play an important role in attracting, optimising and supporting the effective delivery of MLTC research.
