# Supplementary material for: Rising to the challenges of research in multiple long-term conditions: health and social care research ecosystem approaches
Source: BMJ Open. 2025 Sep 14;15(9):e096206. doi: 10.1136/bmjopen-2024-096206 (PMC12434738; doi:10.1136/bmjopen-2024-096206)
Supplement: Supplementary file 1 [file bmjopen-2024-096206.reviewer_comments.pdf]

PEER REVIEW HISTORY

BMJ Open publishes all reviews undertaken for accepted manuscripts. Reviewers are asked to complete a checklist review form and are provided with free text boxes to elaborate on their assessment. These free text comments are reproduced below.

ARTICLE DETAILS

Title (Provisional)

Rising to the challenges of research in multiple long-term conditions: health and social care research ecosystem approaches

Authors

De Soyza, Anthony; Goodwin, Victoria; Roberts, Kate; Masoli, Jane; Mathews, Jacqueline; Wilding, John; Nestor, Gary; Bower, Peter

---

VERSION 1 - REVIEW

**Reviewer**	**1**
**Name**	**Wodchis, Walter**
**Affiliation**	**University of Toronto, Institute of Health Policy Management and Evaluation**
**Date**	**08-Apr-2025**
**COI**	**None**

BMJ open 2024 096206.R1

Thank you for the opportunity to review this paper outlining the NIHR-CRN opportunities to develop and deliver research related to multimorbidity.

Abstract:

There are 3 opportunities to improve the abstract:

1 Consider the expression of the purpose of the article “In this article we share insights” and make it more purposeful :…something like “ The purpose of this article is to share insights from the NIHR…”

2. You use the terms ‘planning, placement and delivery of health and care research’. The term ‘placement’ is not intuitive without explanation, is there a way to better express that term?

3. you state … “is vital to ensure studies are deliverable”. But it’s similarly not clear what the term “deliverable” means.

Article comments:

"Planning MLTC research" . The second paragraph seems to highlight a tension between fidelity to a specific protocol and pragmatism of real world implementation. You might more explicitly call out this tension.

In the second paragraph you note the need for "hybrid models of research" but it is not clear what you mean by this expression of 'hybrid' . Are you referring to hybrid implementation and evaluation research ?

Social care teams involved in this research may have less capability and experience with research but that seems implied but not explicit. Is this your meaning? If so, perhaps be more explicit.

P4. Line 20. "… ensure representation of the range of MTLC types". By "types" are you referring to combinations? Other typologies? It is a bit vague as what "MLTC types" might be and it seems important to the discussion.

Table 1. 3rd type of outcome is "Treatment burden and adverse drug reactions" . these seem to be very different considerations with one being burden and the other adverse events.

8th type is "mental health and social interaction" … but the text speaks to mental and physical health interactions. Seems a mismatch.

9th type. Description is confusing. How is the primary research area unrelated to the intervention?

Table 3. 5th type of intervention uses the word "comorbidome" … I have never heard of that expression ? What does it mean ? I looked it up and it seems to be all the possible comorbidities associated with a condition but your usage is not specific to a particular condition. Perhaps more generic wording as this appears only once and is not widely used.

P 11 paragraph 2 ("The UK based Patient Research Experience…" This paragraph seems to get off track. It starts with the survey but then morphs to delivery of complex interventions and difficulties in delivery of MLTC studies and inclusion of research settings in RDN health and care leadership model. At this point it seems far from the initial introduction. I recommend redrafting this section with greater clarity on the purpose of the text.

In the conclusion I recommend that you draw back to the two main ways that MLTC research can be advanced – through increasing inclusion and explicit consideration of individuals with MLTC in single-disease research and in the focus on MLTC as a focus for research. Both are essential to advance knowledge and improve the evidence base for managing MLTC which is a vexing and complex topic.

**Reviewer**	**2**
**Name**	**Highton, Patrick**
**Affiliation**	**University of Leicester, Cardiovascular Sciences**

**Date**	**10-Apr-2025**
**COI**	**None**

Well written and interesting review. Some minor comments/suggestions:

Page 2 Line 21 – could you define complex MLTC in this instance

Page 2 Line 25 – could you also expand on the differing aetiology of MLTC in younger populations

Page 3 Line 1-2 – could also mention that the external validity of single disease studies with tight inclusion criteria is becoming increasingly limited. It might be good for evidencing efficacy of an intervention, but no use in the real world where most patients will have other conditions

Page 3 Line 37 onwards – could also mention the simple difficulty of choosing an outcome measure that is meaningful (clinically or otherwise) to the heterogenous population of those with MTLCs, and amenable to change via a complex intervention in order to inform a sample size calculation that would be realistically fundable. Perhaps more exploration of co-primary or composite primary outcomes?

Page 10 Lines 12-14 – also issues like lack of trust due to previous experiences, and lack of cultural competency on behalf of research and clinical teams

VERSION 1 - AUTHOR RESPONSE

Reviewer: 1	Abstract:<br>There are 3 opportunities to improve the abstract:<br>1 Consider the expression of the purpose of the article “In this article we share insights” and make it more purposeful :…something like “ The purpose of this article is to share insights from the<br>NIHR…”	1. Please refer to Pg 2, Line 3, we have updated the manuscript as suggested.<br>2. Please refer to Pg2, Line 7&8 and Pg2 Line14&15, we have deleted the term ‘placement’ and added amended the wording to address the points raised.

**Source**	**Comment**	**Response**

	2. You use the terms ‘planning, placement and delivery of health and care research’. The term ‘placement’ is not intuitive without explanation, is there a way to better express that term?<br>3. you state … “is vital to ensure studies are deliverable”. But it’s similarly not clear what the term “deliverable” means.	3. Please refer to Pg2, Line 12-13, we have amended the wording to address the comments raised.
	“Planning MLTC research”. The second paragraph seems to highlight a tension between fidelity to a specific protocol and pragmatism of real world implementation. You might more explicitly call out this tension.	See Pg 3, Line 29-32, we have added additional sentences in this section, to address the points raised.
Reviewer 2	Page 2 Line 21 – could you define complex MLTC in this instance	Please see Pg 2, Line 24 & 25, we have updated the description as follows complex-MLTC (Complex-MLTC: 4 or more conditions)(4)
Reviewer 1	In the second paragraph, you note the need for “hybrid models of research” but it is not clear what you mean by this expression of ‘hybrid”. Are you referring to hybrid implementation and evaluation research? Social care teams involved in this research may have less capability and experience with research but that seems implied but not explicit. Is this your meaning? If so, perhaps be more explicit.	Please see Pg4, L4-10, we have added additional text to address the reviewer’s comments.
Reviewer 1	P4. Line 20. “… ensure representation of the range of MTLC types”. By “types” are you referring to combinations? Other typologies? It is a bit vague as what “MLTC types” might be and it seems important to the discussion.	Please see Pg4, Line 38&39, we have added additional text to address the reviewer’s comments.

Reviewer 1	Table 1. 3rd type of outcome is “Treatment burden and adverse drug reactions” . these seem to be very different considerations with one being burden and the other adverse events.	The table has been updated to split the original row 3 into two separate rows Treatment (or care) burden and Adverse drug reactions.

**Source**	**Comment**	**Response**
	8th type is “mental health and social interaction” … but the text speaks to mental and physical health interactions.<br>Seems a mismatch.<br><br>9th type. Description is confusing. How is the primary research area unrelated to the intervention?	This has been updated. The Type has been updated to include an additional reference to physical health. The description has been updated to highlight potential challenges and the need to ensure the perspectives of patients and service users inform the study design.<br><br>An example has been added. (e.g. home-based nurse-led anxiety counselling leading to signposting to social care<br>support for falls prevention)
	Table 3. 5th type of intervention uses the word “comorbidome” … I have never heard of that expression ? What does it mean ? I looked it up and it seems to be all the possible comorbidities associated with a condition but your usage is not specific to a particular condition. Perhaps more generic wording as this appears only once and is not widely used.	Page 10, we have replaced this with comorbidity.

	P 11 paragraph 2 ("The UK based Patient Research Experience…" This paragraph seems to get off track. It starts with the survey but then morphs to delivery of complex interventions and difficulties in delivery of MLTC studies and inclusion of research settings in RDN health and care leadership model. At this point it seems far from the initial introduction. I recommend redrafting this section with greater clarity on the purpose of the text.	See Pg 13, Line 20 and Pg 14, Line 23-30, we have amended the ordering of this section to address the points raised.
	In the conclusion, I recommend that you draw back to the two main ways that MLTC research can be advanced – through increasing inclusion and explicit consideration of individuals with MLTC in single-disease research and in the focus on MLTC as a focus for research. Both are essential to advance knowledge and	See Pg 14, Line 3-50 and Pg15, -2. We have added a further sentence to the conclusion as per the reviewer's feedback.
**Source**	**Comment**	**Response**
	improve the evidence base for managing MLTC which is a vexing and complex topic.	
Reviewer 2	Page 2 Line 25 – could you also expand on the differing aetiology of MLTC in younger populations	See Pg, 2 Line 29, we have added a further comment on this.
Reviewer 2	Page 3 Line 1-2 – could also mention that the external validity of single disease studies with tight inclusion criteria is becoming increasingly limited. It might be good for evidencing efficacy of an intervention, but no use in the real world where most patients will have other conditions	Please see Pg3, Line 5&6, we have added additional text in response to the reviewer's comments.

Reviewer 2	Page 3 Line 37 onwards – could also mention the simple difficulty of choosing an outcome measure that is meaningful (clinically or otherwise) to the heterogenous population of those with MTLCs, and amenable to change via a complex intervention in order to inform a sample size calculation that would be realistically fundable. Perhaps more exploration of co-primary or composite primary outcomes?	Please see Pg4, Line 14-17, we have updated the section as requested.
Reviewer 2	Page 10 Lines 12-14 – also issues like lack of trust due to previous experiences, and lack of cultural competency on behalf of research and clinical teams	See Page 12, Line 18 & 19, we have added an additional sentence to address this comment.

VERSION 2 - REVIEW

Reviewer 1

Name Wodchis, Walter

Affiliation University of Toronto, Institute of Health Policy Management and Evaluation

Date 24-Jun-2025

COI

The authors have fully addressed the concerns and issues raised.
